# From Balance to Breakdown: striatal PV interneurons in Huntington’s disease and Autism Spectrum Disorder

**DOI:** 10.3389/fncel.2026.1717636

**Published:** 2026-02-23

**Authors:** Mathieu Thabault, Laurie Galvan

**Affiliations:** 1 APC Microbiome Ireland, University College Cork, Cork, Ireland; 2 Department of Pharmacology and Therapeutics, University College Cork, Cork, Ireland; 3 NIMES UNIV, APSY-V, Nîmes, France

**Keywords:** Autism Spectrum Disorders, GABA, Huntington’s disease, parvalbumin interneuron, striatum

## Abstract

Once relegated to the background of striatal circuitry, parvalbumin-expressing interneurons are now emerging as central players in health and disease. Acting as true gatekeepers, striatal PV interneurons are well-described for their role in synchronizing striatal output and balancing excitation and inhibition to sustain coordinated motor and cognitive functions. In this review, we highlight recent advances in understanding their developmental origins, molecular identity, physiological properties, and their roles in striatal function. Furthermore, we examine converging evidence implicating PV interneurons in Huntington’s disease and Autism Spectrum Disorder, where their structural, molecular, and functional alterations position them at the intersection of neurodegenerative and psychiatric research.

## 1 Introduction

The basal ganglia, also referred to as central grey nuclei, are a group of subcortical structures remarkably conserved through evolution: the striatum, the globus pallidus, the subthalamic nucleus and the substantia nigra. A true gateway to the basal ganglia network, the striatum takes its name from the Latin ‘Corpus Striatum’, literally “striate body” referring to the numerous white matter tracts that cross this structure. Long considered to be responsible for only primitive functions such as movement control, the striatum is nowadays well-known for its role in higher brain functions, i.e., fine motor coordination, associative learning, decision making and reward system, all of which are supported by functionally distinct subterritories such as the dorsolateral striatum (DLS), dorsomedial striatum (DMS), and ventral striatum. Unlike the cortex or the hippocampus for example, the striatum is organized in a non-laminar way. Instead, it is structured in two interdigitated compartment: the striosome and the matrix. First described in the late 1970’s (Graybiel and Ragsdale, 1978), this organization is thought to underlie parallel functional streams within the striatum [for review: (Prager and Plotkin, 2019)]. Incidentally, this organization prevents the striatal projection neurons (SPNs), from integrating inputs sequentially and requires the striatal microenvironment to be highly responsive, through striatal interneuron diversity of cell-types, activities and roles, which has been previously extensively described [for review: (Tepper et al., 2010)].

**Figure 1:**
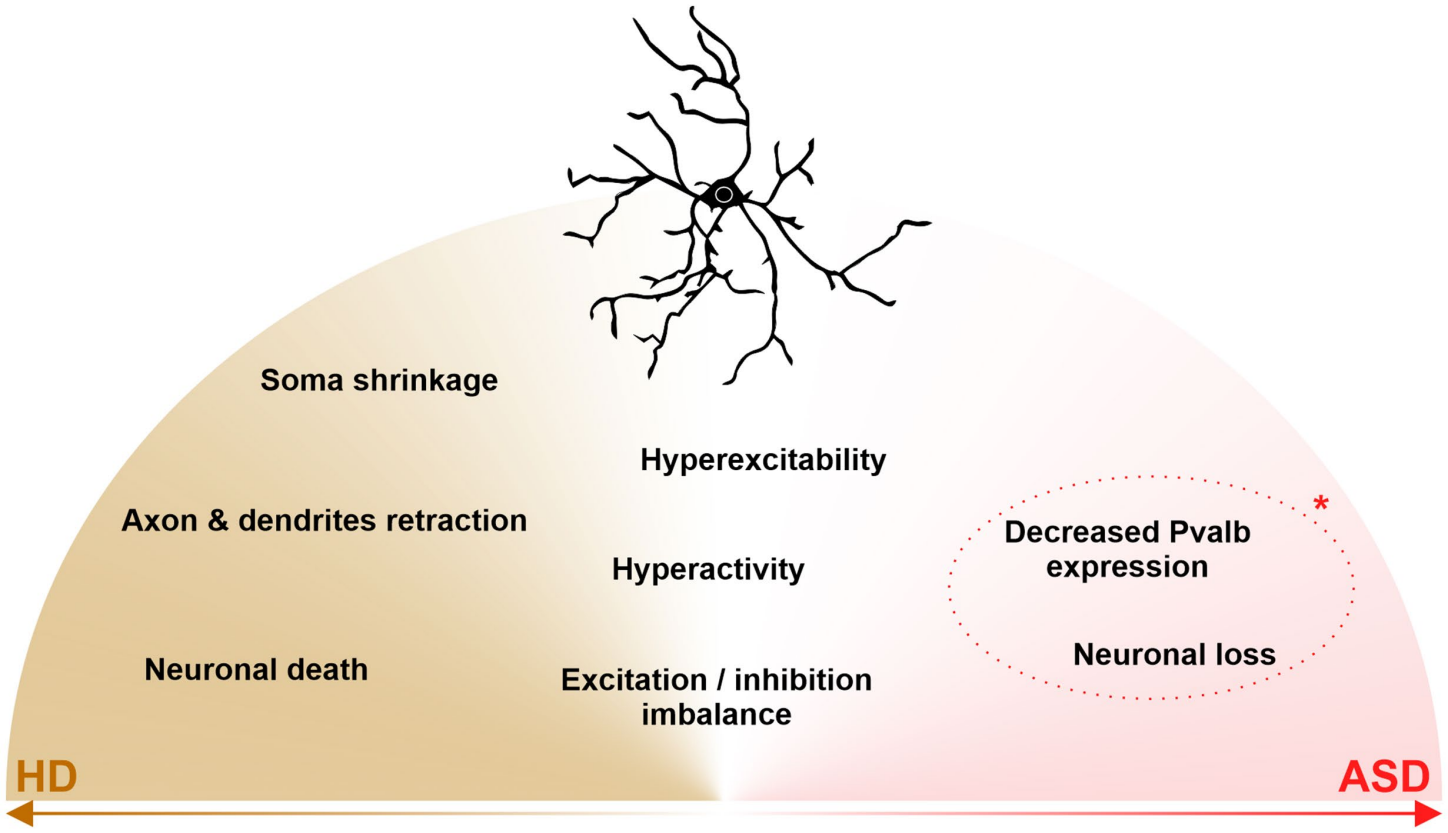
Shared and disease-specific alterations and dysfunctions of striatal parvalbumin interneurons in Huntington’s disease (HD) and Autism Spectrum Disorder (ASD). In HD (left, brown), striatal PV interneurons show neurodegeneration, somal shrinkage, and axonal/dendritic retraction. In ASD (right, pink), striatal PV interneurons are decreased in number, although it’s a current key debate (dotted line, *) as to whether the reduction of PV interneurons is due to a neuronal loss or decreased PVabl expression. In the middle (white), striatal PV interneurons undergo excitation/inhibition imbalance and display neuronal hyperactivity and hyperexcitability, all three features being shared between HD and ASD.

In this mini-review, we provide a state-of-the-art definition of striatal parvalbumin-expressing (PV) interneurons and describe their central role in striatal functions through the scope of their involvement in neurodegenerative disorders and psychiatric disorders (see Figure 1).

### 1.1 Striatal parvalbumin interneurons

Parvalbumin-expressing neurons have been extensively studied in cortical and hippocampal circuits, where they comprise a highly heterogeneous population with diverse molecular, morphological, and functional properties (Hao et al., 2021). Striatal PV interneurons form a comparatively, yet discussed, less heterogeneous population whose principal role is to provide rapid and powerful feedforward inhibition onto striatal projection neurons (SPNs). Given that SPNs constitute the sole output of the striatum, PV interneuron-mediated inhibition represents a critical mechanism for controlling striatal information flow within the basal ganglia, such that dysfunction of this neuronal population is expected to profoundly impact striatal-dependent pathologies.

#### 1.1.1 Neurodevelopmental trajectories and maturation

Striatal PV interneurons originate predominantly during embryonic development from the preoptic area and the septal neuroepithelium, with more modest contributions from the caudal ganglionic eminence (Knowles et al., 2021). Within the septal-derived medial ganglionic eminence (MGE), a strong cell fate specificity is observed, with basal intermediate progenitors largely contributing to the generation of PV interneurons. This early specification of neuronal identity relies on a complex transcriptional network, mainly transcription factors such as Nkx2.1, Lhx6, and Lhx8, which are crucial for the proper maturation of PV + GABAergic interneurons. Following their genesis, MGE-derived PV interneurons undergo extensive tangential migration toward the striatum, guided by a combination of the attractive Neuregulin 1 (Nrg1)/ErbB4 system and repulsive EphB/ephrinB signaling (Knowles et al., 2021). The sustained expression of Nkx2.1 in striatal-destined cells functions as a postmitotic “lock,” preventing their migration into the cortex. Once settled in the striatum, PV interneurons are not homogeneously distributed. They are particularly enriched in the dorsolateral striatum (DLS) (Rotariu et al., 2025) and show higher density within the striatal matrix and at peristriosomal boundaries.

#### 1.1.2 Molecular identity and electrophysiological properties

The molecular identity of the striatal PV interneurons comes from the strong expression of the Pvalb gene, coding for the parvalbumin protein. The parvalbumin protein is a calcium buffer, i.e., chelates Ca2 + ions in the neuronal cytoplasm, and regulates calcium influx and signaling [for review: (Schwaller, 2020)]. Such fine-tuned calcium signaling underlies the characteristic electrophysiological features of striatal PV interneurons. Striatal PV interneurons exhibit fast spiking activity, characterized by high-frequency, short-duration discharges (Kawaguchi, 1993; Kita et al., 1990). Interconnected through gap junctions, they form electrotonic coupling networks that promote local synchronization. *In vivo*, or under high-frequency activation, such synchronization generates and sustains gamma oscillations across cortico-striatal circuits (Berke et al., 2004). Cortical desynchronization for example, enhances PV interneuron activity, leading to strong SPN inhibition via decreased excitation and increased inhibition. Their shorter response latencies further suggest a role in temporally constraining SPN discharges (Mallet et al., 2005). Striatal PV interneuron excitability is finely tuned by neuromodulators: dopamine enhances activity via D5 receptor activation, acetylcholine acts through non-desensitizing nicotinic receptors, and endocannabinoids modulate their function via CB1 receptors. PV interneurons exert powerful feedforward inhibition on SPNs, forming numerous perisomatic and dendritic synapses, highly effective in preventing SPN action potential generation (Koós and Tepper, 1999; Mallet et al., 2005). A single PV interneuron can contact 135–541 SPNs, while each SPN receives inhibitory input from 4 to 27 PV interneurons (Koós and Tepper, 1999). Unlike SPNs, which integrate many weak inputs, PV interneurons receive strong excitatory cortical and thalamic inputs from relatively few but potent afferents, enabling rapid action potential generation without extensive summation. They also receive inhibitory input from other interneurons and globus pallidus neurons. Together, these anatomical and synaptic features place striatal PV interneurons in a key position to provide fast, powerful feedforward inhibition and to precisely regulate the timing and synchrony of SPN activity within striatal circuits.

#### 1.1.3 Molecular heterogeneity and plasticity of striatal PV interneurons

Long considered as one single homogenous population, recent transcriptomic studies started to unveil some heterogeneity in striatal interneuron population. While some data suggest that striatal PV interneurons in fact consist of at least two subpopulation based on the expression of the Pthlh gene (Bengtsson Gonzales et al., 2020), other data suggest that PV-fast spiking interneurons might be a subpopulation of Pthlh-expressing interneurons (Muñoz-Manchado et al., 2018) This heterogeneity was proposed to distinguish three cell types: “classical” fast-spiking (FS) and “fast-spiking-like” PV-expressing interneurons (FSL), and Pthlh-expressing/PV-non expressing FLS interneurons. These subtypes were reported to display distinct spatial organization, particularly in the DLS, where FS cells were more abundant and displayed more complex axonal arborization than in the dorsomedial striatum for example. The authors suggested that FS cells in the DLS may be better suited for processing high-intensity cortical inputs. However, while these transcriptomic distinctions add granularity to interneuron classification, they do not yet provide compelling evidence for functionally discrete PV interneuron subclasses other than spatial organization, and their primary consequence may be a reorganization of terminology rather than a conceptual shift. A recent study (Naon et al., 2025) provided, for the first time, a comprehensive translatome of dorsal striatal PV interneurons, which had previously remained poorly characterized at the molecular level. Using the RiboTag technique in Pvalb-Cre mice, the authors identified over 2,700 transcripts significantly enriched in these interneurons (enrichment factor > 1.5). Moreover, the authors showed that in an operant conditioning paradigm involving food rewards of different values, more than 100 genes were differentially enriched in dorsal striatal PV interneurons depending on reward type (standard vs. highly appetitive). These findings confirmed the expression of classical PV interneuron markers, while also revealing a distinct molecular signature differentiating dorsal striatal PV interneurons from those of the nucleus accumbens (NAc). Gene ontology analysis indicated a strong enrichment of genes involved in various neurotransmission systems, but also in cell adhesion molecules and extracellular matrix components.

Interestingly, PV interneurons are surrounded by perineuronal nets (PNNs), specialized extracellular matrix structures that surround inhibitory neurons, particularly this type. PNNs stabilize synaptic connections and regulate neuronal plasticity (Auer et al., 2025; Mueller-Buehl et al., 2022). Their distribution varies across brain and spinal cord regions (Lupori et al., 2023; Seeger et al., 1994), and their developmental emergence progresses at region-specific rates (Adams et al., 2001; Bruckner et al., 2000). In the striatum, PNNs appear in the matrix during the second postnatal week, coinciding with PV interneuron maturation and regional functional transition, restricting synaptic modifications while preserving circuit integrity (Lee et al., 2018). Although this encapsulation limits synaptic remodeling in adulthood (Carceller et al., 2020; Sánchez-Ventura et al., 2022; Wingert and Sorg, 2021) recent studies show that enzymatic degradation of PNNs can reopen critical periods of plasticity (Fino et al., 2018; Li et al., 2024). These findings offer therapeutic perspectives for neurological disorders characterized by impaired plasticity but raise critical questions regarding the balance between synaptic flexibility and the stability ensured by PNNs.

### 1.2 Striatal parvalbumin interneurons in pathology

Although Huntington’s disease (HD) and autism spectrum disorder (ASD) are traditionally classified as neurodegenerative and neurodevelopmental disorders, respectively, accumulating evidence challenges this strict dichotomy and points to shared circuit-level vulnerabilities across the lifespan. Striatal parvalbumin (PV) interneurons are ideally positioned to illuminate these convergences, as they play a critical role in shaping striatal output through fast, feedforward inhibition and in regulating network synchrony during both development and adulthood. In HD, beyond the well-characterized progressive degeneration of striatal neurons, growing evidence supports a neurodevelopmental component of the disease (Van Der Plas et al., 2020), with mutant huntingtin disrupting early developmental processes and circuit maturation long before described neurodegeneration. Conversely, ASD is classically viewed as a disorder of early brain development, but exhibits pronounced age-dependent changes, including alterations in striatal circuitry and inhibitory function that emerge or evolve during adolescence and adulthood (Thabault et al., 2023). Together, these observations suggest that dysfunction of striatal PV interneurons may reflect not only disease-specific mechanisms but also shared principles of circuit instability, maladaptive plasticity, and impaired inhibitory control that manifest differently across developmental and degenerative trajectories.

#### 1.2.1 Huntington’s disease

Huntington’s disease (HD) is an autosomal dominant neurodegenerative disorder caused by a mutation in the HTT gene on chromosome 4p16.3, encoding the protein huntingtin (HTT). The mutation involves an expansion of a CAG trinucleotide repeat in exon 1, resulting in a polyglutamine (polyQ) expansion within the mutant protein (mHTT). Clinically, HD manifests as a triad of motor (chorea), cognitive, and psychiatric symptoms, with variable onset and severity among patients [for review: (Koehler and Jennekens, 2008; Macdonald, 1993)].

HD is a progressive disorder with neuropathological stages classically defined by DiFiglia et al. (1997) and Vonsattel et al. (1985), who described five grades (0–4) of increasing severity, based on macroscopic and microscopic criteria closely correlated with clinical status. Until the 2010s, it was widely believed that only SPNs were vulnerable, while striatal interneurons were thought to be “relatively spared.” To better understand interneuron involvement, particularly PV interneurons, a symptom-based classification proposed by Professor Faull has been employed as an alternative to Vonsattel’s anatomical staging. This approach stratifies patients according to their dominant symptomatology: “mood disorder” (cognitive/psychiatric), “motor,” or “mixed,” with the aim of identifying common molecular mechanisms underlying variability in symptom intensity and profiles across patients. Using this approach, Faull’s group examined postmortem brains from HD patients across five cortical regions (Kim et al., 2014; Mehrabi et al., 2016). In the superior frontal cortex, HD patients exhibited substantial reductions in CB-, PV-, and CR-expressing interneurons, with distinct patterns across motor-dominant, mood-dominant, and mixed clinical subtypes. Soma volumes of CB and PV interneurons were also reduced. In the superior parietal cortex, PV interneurons were significantly reduced in HD patients compared to controls, and no significant correlation was found between cortical interneuron loss and Vonsattel neuropathological grade (Mehrabi et al., 2016). Such cortical observations prompted further examination of interneuron vulnerability within the striatum.

Striatal PV interneurons exhibit progressive degeneration in patients, particularly within the motor putamen, where their loss correlates with the onset and worsening of dystonia (Reiner and Deng, 2018). In mouse models of HD, major cell loss is not observed; instead, PV interneurons exhibit soma shrinkage and retraction of dendritic and axonal projections (Holley et al., 2019a; Reiner and Deng, 2018). Unfortunately, many earlier studies relied exclusively on stereological counts, lacking data on dendritic and axonal integrity. As suggested here however, an interneuron with a preserved soma but without projections is likely incapable of fulfilling its physiological role in network regulation, as evidenced by the fact that neurotransmission deficits have been observed in preclinical studies. Indeed, in the Q175 mouse model of Huntington’s disease, PV interneurons (FSI) display significant morphological degeneration at symptomatic stages (12 months). These alterations include reduced soma area (90.3 ± 5.0 μm^2^ in Q175 vs. 108.3 ± 5.9 μm^2^ in WT) and decreased dendritic complexity (Holley et al., 2019b). Electrophysiologically, FSIs from Q175 mice exhibit decreased membrane capacitance, increased input resistance, and more depolarized resting membrane potentials (RMP: −69.8 ± 2.1 mV in Q175 vs. −76.7 ± 1.2 mV in WT) (Holley et al., 2019b). These changes lead to enhanced intrinsic excitability, characterized by a reduced rheobase and an increased number of action potentials evoked by current injections (Holley et al., 2019b). Paradoxically, these interneurons also receive significantly fewer excitatory synaptic inputs at symptomatic stages, resulting in a decreased excitation–inhibition ratio (2.8 ± 0.4 in Q175 vs. 4.9 ± 0.7 in WT; *p* = 0.021). The morphological degeneration of FSIs, rather than causing hypoexcitability, appears to trigger a compensatory or pathological increase in intrinsic excitability. This hyperexcitability, combined with reduced excitatory drive, suggests that FSIs may integrate cortical inputs less effectively (due to diminished dendritic arborization and fewer excitatory inputs), yet maintain the capacity to deliver strong, but potentially dysregulated, feedforward inhibition onto SPNs.

The observed loss of PV interneurons in human HD patients (Holley et al., 2019b) further supports the notion that FSI dysfunction represents a critical component of the disease, potentially contributing to motor symptoms such as dystonia, through impaired regulation of striatal output. In support of this, optogenetic stimulation of PV-expressing interneurons in R6/2 mice induces significantly larger and faster GABAergic currents in SPNs, indicating enhanced feedforward inhibition (Cepeda et al., 2013). While reduced parvalbumin expression could contribute to under-detection of PV interneurons, the convergence of morphological, electrophysiological, and disease-stage–dependent evidence, together with the absence of clear demonstrations of isolated PV downregulation, supports the prevailing view that PV interneurons undergo progressive degeneration and loss in HD.

Together, these findings highlight striatal PV interneurons as key modulators of pathological striatal activity in HD, linking cellular and circuit-level alterations to behavioral dysfunction, and raising the possibility that similar inhibitory circuit mechanisms may be disrupted in other disorders affecting striatal function.

#### 1.2.2 Autism Spectrum Disorder

Autism Spectrum Disorder (ASD) is a set of neurodevelopmental disorders defined as a combination of symptoms that are grouped into two domains: (i) Persistent deficits in social communication and social interaction across multiple contexts and (ii) Restricted, repetitive patterns of behavior, interests, or activities (American Psychiatric Association, 2022). ASD etiology consists of the interplay between genetic, epigenetic, infectious and pharmacological factors, which may act at different time points from conception to birth and early-life. Some ASD cases are even idiopathic, i.e., without an identified and/or defined cause, highlighting the high complexity of ASD etiology. Through decades of ASD studies, several hypotheses have emerged to explain ASD etiology and/or pathophysiology. One of the most widely accepted hypotheses was first proposed in 2003, when Rubenstein and Merzenich (2003) published the excitation/inhibition imbalance hypothesis of ASD. While healthy brain networks rely on a fine balance between excitatory and inhibitory inputs, ASD patients often display deficits or an excess of either form of neurotransmission in various brain structures, irrespective of the cause.

Since its formulation, this excitation/inhibition imbalance hypothesis has been investigated across brain structures, among which the striatum gained particular attention due to its role in motor, associative and cognitive systems [for review: (Fuccillo, 2016; Thabault et al., 2022)]. The first evidence for striatal involvement in ASD pathophysiology dates back to the late 1980s/early 1990s, when neuroimaging studies revealed anatomical alterations of its structures (Aylward et al., 1991; Creasey et al., 1986; Reiss et al., 1993). Subsequent MRI studies conducted in the 2000s and 2010s reported hypertrophy of the putamen, caudate nucleus, and Nucleus Accumbens in ASD (Hollander et al., 2005; Rojas et al., 2006). Similar anatomical changes in the striatum have also been observed in several ASD animal models. These anatomical abnormalities are accompanied by cellular alterations (Ahmed et al., 2023). In humans, a reduction in neuronal density has been described despite the hypertrophy of striatal nuclei, with decreases of 15% in the Nucleus Accumbens and 13% in the putamen. In animal models, although no significant changes in overall striatal neuronal density have been reported, several specific neuronal populations are affected [for review: (Evans et al., 2024)].

Among them, the striatal PV interneurons appear to be likely candidates that contribute to ASD pathophysiology (Filice et al., 2020). First, when lacking the expression of the Pvalb gene, mice exhibit core ASD symptoms such as alterations in developmental features, social impairments and motor stereotypies (Wöhr et al., 2015), although this study does not provide insights about striatal PV interneurons specifically. Then, a depletion study from 2017 (Rapanelli et al., 2017) addressed this question partially. Performing conjoint targeted cholinergic and PV interneuron depletion in the dorsal striatum, the authors showed that male mice begin to display autism-like behavioral abnormalities, while females do not seem to exhibit phenotypic changes. Notably, the authors also performed targeted depletion of striatal PV interneurons but did not report autism-like behavior in this case. However, this result should be interpreted with caution as this part of the study is (i) limited to adulthood depletion of striatal PV interneurons, (ii) with a small sample size (*n* = 5), and (iii) only looked at the stereotypic time as the sum of grooming and digging behavior, omitting stereotyped behavior frequency and not distinguishing syntactic grooming behavior (extensively described in Lamothe et al., 2023) from short, stereotyped grooming.

Interestingly, striatal PV interneurons are also reported to be affected in canonical models of ASD. Male rats exposed to valproic acid (VPA) in utero show increased grooming behavior, associated with a decrease of PV and cholinergic interneuron number, particularly in the dorsal striatum (Ibáñez-Sandoval et al., 2024). Interestingly, mice exposed in utero to VPA display decreased expression levels of Pvalb in the striatum, but a normal number of PV neurons (Lauber et al., 2016). Similar results have been reported from Shank1−/− and Shank3B−/− mice (Filice et al., 2016) and from Cntnap2−/− mice (Lauber et al., 2018), hinting toward probable points of convergence. In the aforementioned studies, except Ibáñez-Sandoval and colleagues, authors co-labeled PV and perineuronal nets (PNN) surrounding PV interneurons, to reveal that although PV labeling could be reduced by up to 60%, the number of PNN + neurons remained similar to WT or untreated mice. This suggests that PV interneuron maturation and migration in the dorsal striatum is maintained in ASD, pointing towards loss-of-function rather than a loss of PV interneurons. In Shank3B−/− and Cntnap2−/− mice, the authors also reported deregulation of Hcn1 and Hcn4 genes expression respectively, suggesting altered intrinsic activity of striatal PV interneurons. In Cntnap2−/− mice, a recent study (Thabault et al., 2024) demonstrated that striatal PV interneurons exhibit hyperactivity and hyperexcitability. Specifically, authors showed that, in brain sections, 87% of recorded striatal PV interneurons were firing spontaneously, while they have been shown to be silent in similar conditions [for review: (Tepper et al., 2010)]. In addition, striatal PV interneurons that were initially silent exhibited increased excitability as indicated by an enhanced input–output curve. More importantly, the authors performed chronic DREADD silencing of striatal PV interneurons and revealed a phenotypic restoration of stereotyped behavior, as measured by excessive grooming behavior.

Together, these findings provide convergent evidence that ASD-associated alterations in the striatum involve a functional dysregulation of PV interneuron excitability, which is sufficient to drive stereotyped behaviors and represents a mechanistically relevant target for phenotypic rescue.

### 1.3 Discussion

Converging evidence indicates that striatal PV interneurons are dysfunctional across a wide range of neuropsychiatric conditions, including Huntington’s disease and ASD, as discussed in this review. Although these disorders differ in etiology and time course, both are characterized by striatal circuit dysfunction and impaired control of motor and repetitive behaviors, suggesting convergence at the level of inhibitory network regulation. Striatal PV interneurons, which play a critical role in shaping the timing and synchronization of medium spiny neuron activity, may represent a particularly vulnerable node within these circuits. Dysfunction of these cells is therefore well positioned to produce shared phenotypic features across neurodegenerative and neurodevelopmental conditions. Taken together, the accumulating evidence highlights striatal PV interneurons as promising therapeutic targets in the coming years.
