# Correction: From Balance to Breakdown: striatal PV interneurons in Huntington's disease and Autism Spectrum Disorder

**DOI:** 10.3389/fncel.2026.1872802

**Published:** 2026-05-27

**Authors:** Mathieu Thabault, Laurie Galvan

**Affiliations:** 1 APC Microbiome Ireland, University College Cork, Cork, Ireland; 2 Department of Pharmacology and Therapeutics, University College Cork, Cork, Ireland; 3 NIMES UNIV, APSY-V, Nîmes, France

**Keywords:** Autism Spectrum Disorders, GABA, Huntington's disease, parvalbumin interneuron, striatum

The reference 41 was erroneously written as

41. Naon C, Castell L, Thirard S, Moreno M, Rialle S, Goetz E, et al. (2025). Dorsal Striatum Parvalbumin interneurons translatome unveiled. *AJNR Am. J. Neuroradiol*.12, 1217–1222. doi: 10.1101/2025.06.16.659856

It should be

41. Naon, C., Castell, L., Thirard, S., Moreno, M., Rialle, S., Goetz, E., et al. (2025). Translatome of dorsal striatum parvalbumin interneurons revisited: insights across diverse experimental paradigms. *Front. Cell. Neurosci*. 19:1648461. doi: 10.3389/fncel.2025.1648461

The original version of this article has been updated.

